# Phage‐Mediated Presentation of a Conserved HA2 Epitope From Influenza A Virus Elicits Significant IgY Antibody Responses in Broiler Chickens

**DOI:** 10.1002/vms3.70780

**Published:** 2026-01-14

**Authors:** Zinat Lotfi, Mehdi Golchin, Mohammad Ali Shamshirgaran

**Affiliations:** ^1^ Department of Pathobiology, Faculty of Veterinary Medicine Shahid Bahonar University of Kerman Kerman Iran

**Keywords:** birds, HA2 peptide, hybrid phage, influenza virus, vaccine

## Abstract

Peptides alone often exhibit limited immunogenicity, necessitating the development of effective antigen delivery systems to facilitate recognition and presentation, ultimately eliciting robust immune responses and activating T and B lymphocytes. Filamentous bacteriophages, such as M13, are recognized as efficient platforms for peptide expression and presentation via their capsid surfaces. The conserved amino acid sequence HA2 1–9 (GLFGAIAGF) from the haemagglutinin (HA) transmembrane protein of Influenza A virus (IAV) has been identified as a promising immunogen for eliciting broad‐spectrum immune responses against diverse IAV strains. In this study, the N‐terminal fragment of Protein VIII from M13 phage was genetically engineered to express the conserved HA2 1–9 sequence. High‐level expression of the HA2 peptide on the phage surface was confirmed via immunoblotting analysis. Birds were intramuscularly vaccinated with the recombinant M13 phage displaying the HA2 peptide and subsequently challenged intranasally with the H9N2 IAV subtype. The results demonstrated that the GLFGAIAGF peptide elicited specific immunoglobulin Y (IgY) antibody responses against the HA2 peptide in birds. However, vaccination did not lead to a significant reduction in the shedding of H9N2 virus in the trachea and cloaca. This study highlights the potential of phage display platforms for antigen expression and immune activation. While the conserved GLFGAIAGF epitope successfully induced specific IgY responses, the limited efficacy in reducing viral shedding underscores the need for further optimization of the vaccination regimen, as well as investigation of alternative delivery routes, such as intranasal or oral administration, to enhance protective efficacy.

## Graphical abstract

The conserved region of HA2 peptide of Influenza A virus was expressed on the surface of Protein VIII from M13 phage. Immunization of broiler chickens by this hybrid phage showed high levels of antibody responses, highlighting its potential as a promising tool for universal vaccine development against Influenza A virus.

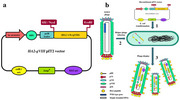

**Abbreviations**
BSA: bovine serum albumin
Com‐C: birds vaccinated with commercial influenza vaccine and challenged
CyaA: detoxified adenylate cyclase toxin
*E. coli*
: *Escherichia coli*
EID 50: embryo infectious dose 50
ELISA: enzyme‐linked immunosorbent assay
GST: glutathione s‐transferase
HA: hemagglutinin
HA2‐C: birds vaccinated with hybrid phage and challenged
HRP: horseradish peroxidase
IAV: influenza A viruses
IgA: immunoglobulin A
IgG: immunoglobulin G
IgY: immunoglobulin Y
IVL: influenza virus‐like particles
KLH: Keyhole Limpet hemocyanin
*L. casei*
: *Lactobacillus casei*
*L. delbrueckii*
: *Lactobacillus delbrueckii*
*L. plantarum*
: *Lactobacillus plantarum*
M13‐C: birds received wild‐type phage and challenged
M2e: matrix protein 2 ectodomain
NA: neuraminidase
NV‐C: birds received PBS and challenged
NV‐NC: birds received PBS and not challenged
PBS: phosphate‐buffered saline
PBST: phosphate‐buffered saline with Tween 20
PCR: polymerase chain reaction
qPCR: quantitative real‐time PCR
*S. cerevisiae*
: *Saccharomyces cerevisiae*
scFv: single‐chain fragment variable
SDS‐PAGE: sodium dodecyl‐sulphate polyacrylamide gel electrophoresis
SPF: specific pathogen‐free
TMB: 3, 3′, 5, 5′ tetramethylbenzidine

## 1 Introduction

Influenza A viruses (IAVs) are recognized as significant etiological agents of respiratory diseases in both human and animal populations (Varečková et al. 2013). Although vaccines utilizing haemagglutinin (HA) and neuraminidase (NA) surface glycoproteins have been employed as the predominant method to address the influenza virus, the successful prevention and management of the associated disease continues to present a challenging obstacle (Margine et al. 2013; Bo et al. 2019). Fragmentation of the influenza virus genome, accompanied by partial and overall changes in antigenicity, arises from point mutations and genetic rearrangements occurring between co‐infecting influenza viruses within a single cell (Pérez‐Losada et al. 2015). Through these mechanisms, the sequence of genes encoding the HA and NA proteins undergoes modifications, leading to the emergence of novel strains of the influenza virus (Han et al. 2023). New strains of the virus have arisen as a significant concern for worldwide public health, giving rise to the manifestation of widespread epidemics or even global pandemics (Margine et al. 2013). To address this, vaccines containing HA and NA antigens must be updated annually, based on ongoing surveillance of circulating influenza strains and their antigenic profiles (Bo et al. 2019).

The HA protein, a significant spike glycoprotein of the Influenza virus, is comprised of HA1 (∼324 amino acids) and HA2 (∼222 amino acids) subunits linked by disulfide bonds, enabling their interaction with surface sialic acid receptors of the host cell and facilitating the crucial process of virus entry into the host cell (Bo et al. 2019). The HA2 subunit exhibits greater antigenic stability than HA1 (Gong et al. 2016). The HA trimer stem or the N‐terminal segment of HA2 is the ‘fusion fragment’ (Varečková et al. 2013). The conserved stretch of approximately 20 amino acid residues found in the IAVs subtypes, is a predominantly intrinsically hydrophobic sequence located at the encoded C‐terminus of the HA protein (Rafalski et al. 1991; Yang et al. 2018). Studies have provided evidence indicating that HA2 sequence GLFGAIAGF (Residues 1–9) exhibits conservation and an exceptionally high degree of antigenicity (Du et al. 2010). By employing this specific sequence, it is feasible to produce a new generation of vaccines encompassing safeguarded peptides, thereby effectively addressing the problem of drifting or shifting IAVs (Wei et al. 2020).

According to the significantly reduced immunogenic potential of the corresponding sequence, there are low levels of antibodies produced against this region during typical antibody production processes (Tomčíková and Varečková 2019). Therefore, mere protection measures are inadequate in mitigating the impact of severe infections caused by novel strains of IAV (Margine et al. 2013). Findings suggest that HA2 induces specific T‐cell and B‐cell immunity. The inhibition of fusion ability or virus replication by HA2‐specific antibodies, along with their inter‐subtype reactivity, highlights HA2 as an immunogen due to the property of eliciting protective immune responses against various influenza virus strains (Varečková et al. 2013). The investigators have been compelled to enhance the immune properties of HA2 peptide by means of conjugation with protein carriers due to the remarkably weak immunogenicity of HA2 peptide in IAV infection, resulting in their low specific antibody induction. These carriers include Keyhole Limpet haemocyanin (KLH) (T. T. Wang et al. 2010), glutathione s‐transferase (GST) (Golchin et al. 2018), influenza virus‐like particles (IVL) (S. Chen et al. 2015), norovirus P and adenovirus (Gong et al. 2016), detoxified adenylate cyclase toxin (CyaA) developed by *Bordetella pertussis* (Staneková et al. 2013), *Lactobacillus plantarum* (Yang et al. 2017, Yang et al. 2018; Bo et al. 2019), phospholipid vesicles (Rafalski et al. 1991).

Several peptide display systems, including bacterial, yeast and phage display platforms, have been utilized for the development of vaccines and therapeutic antibodies against various infectious diseases in poultry (Y. Li et al. 2002, T. Li et al. 2015; Ju et al. 2021; Guo et al. 2024; Shamshirgaran and Golchin 2025a). Antigen display for IAV has been achieved using bacterial systems such as *L. plantarum* (Jiang et al. 2017; Yang et al. 2017; Bo et al. 2019; Niu et al. 2021) and yeast systems such as *Saccharomyces cerevisiae* (Xie et al. 2023; H. Zhang, Li, et al. 2023; H. Zhang, Xie, et al. 2023). Although these platforms are effective for surface protein expression, their library construction is generally more complex and time‐consuming than phage‐based systems (Grabowski et al. 2023). In contrast, phage display provides several unique advantages. It enables the presentation of a broader diversity of peptide variants on the virion surface and allows the generation of more extensive clone libraries compared with bacterial or yeast display approaches (Jaroszewicz et al. 2022). Moreover, display on the major coat protein pVIII facilitates the presentation of thousands of peptide copies on a single phage particle, and this high valency is strongly associated with enhanced immunogenicity (Bratkovič 2010). Additional advantages of filamentous phages that further enhance their potential for vaccine development include their capacity to be produced at exceptionally high titres, reaching up to 10^14^ phages/mL (Jaroszewicz et al. 2022), and their remarkable stability under harsh physicochemical conditions, such as variations in pH and temperature (González‐Mora et al. 2020). The utilization of filamentous phages, specifically M13, fl and fd, as carrier platforms presents notable advantages compared to animal viruses, leading to an enhanced immune response against foreign peptides (Jaroszewicz et al. 2022). The capsid of the filamentous phage, such as M13, consists of a major protein (VIII) and several minor proteins (III, VI, VII and IX) (Jaroszewicz et al. 2022). Within each M13 phage, there are about 2700 pVIII protein copies, enabling the expression of a diverse range of foreign immunogens close to the N‐terminal fragment of the gpVIII protein and facilitating the generation of hybrid phages with the desired peptide epitope incorporated into the major pVIII protein (Jaroszewicz et al. 2022; Grabowski et al. 2023). This study aimed to construct a hybrid phage capable of displaying the HA2 (1–9) IAVs peptide by genetically fusing it to the N‐terminal segment of the pVIII protein. Subsequently, we investigated the efficacy of the hybrid phage as a potential vaccine candidate in stimulating humoral immune responses and reducing the shedding of viruses in broiler chickens.

## 2 Materials and Methods

### 2.1 Bacterial Strain and Growth Conditions

The TG1 strain of *Escherichia coli* was obtained from GeneService (Cambridge, UK), using the Tomlinson I and J single‐chain fragment variable (scFv) libraries. The bacteria were cultivated under aerobic conditions, either in 2xTY broth or on TYE medium, overnight at a temperature of 37°C. Ampicillin (Biobasic, Canada) was added (100 mg/L) whenever needed.

### 2.2 Construction of the Recombinant pIT2 Vector

A gene encompassing the N‐terminal epitope of HA2 (GLFGAIAGF, 1–9), which is universally preserved across all subtypes of IAVs, was genetically engineered. Furthermore, the gVIII gene derived from M13 phages was specifically engineered and synthesized using (5′‐CTTATACCATGGCCGGTCTGTTTGGTGCCATTGCCGGCTTCGAGGGTGACGACCCCGCAAAAG‐3′) and (5′‐ACTCAGAATTCTTATCAGCTTGCTTTCGAGG‐3′) primers. The polymerase chain reaction (PCR) amplification was conducted using the following thermal conditions: an initial denaturation step (5 min/94°C), followed by 35 cycles, each comprising a denaturation step (30 s/94°C), an annealing step (45 s/55°C) and an extension step (1 min/72°C). An additional extension step was performed at 72°C for 5 min. The fragments were then electrophoresed and visualized in a 1% agarose gel.

Subsequently, the digestion of the *HA2‐gVIII* fusion gene and the pIT2 phagemid vector was conducted using the respective restriction enzymes, NcoI and EcoRI (Jena Bioscience, Germany). The *HA2‐gVIII* gene was then purified from the electrophoresis gel, followed by cloning into the pIT2 vector. To confirm the cloning procedure, the constructed recombinant *HA2 (1–9)‐gVIII* pIT2 vector underwent sequencing analysis (Macrogen, Korea) using LMB3 primer (5′CAGGAAACAGCTATGAC3′).

### 2.3 Transformation of the HA2‐gVIII Fusion Gene Into *E. coli*

The recombinant pIT2 vector containing *HA2 (1–9)‐gVIII* fusion gene was introduced into *E. coli* TG1 competent cells through the standard heat shock method (Chan et al. 2013). Briefly, the recombinant pIT2 vector was gently mixed with *E. coli* TG1 competent cells, followed by incubation on ice for 30 min. The resultant mixture was then incubated at 42°C for 90 s. Following the addition of SOC medium to the mixture and subsequent incubation (37°C for 1 h), the bacteria were grown for 24 h at 37°C on ampicillin‐supplemented (100 mg/L) TYE medium containing 4% glucose. The presence of the fusion gene was analysed by screening of the colonies and employing the PCR method described earlier.

### 2.4 Recombinant HA2‐gVIII Protein Expression

A single colony of *E. coli* TG1 harbouring the HA2‐*gVIII*‐pIT2 vector was cultured overnight at 37°C in 2xTY broth supplemented with ampicillin and 4% w/v glucose. The next day, 1% of the culture was utilized to inoculate 250 mL of fresh ampicillin‐supplemented 2xTY medium containing 4% w/v glucose. The culture was then incubated at 37°C with shaking at 250 rpm. When the cells reached OD_600 _= 0.4, 1 × 10^12^, KM13 helper phages were added, followed by incubation (at 37°C for 1 h) without shaking to permit phage entrance to the cells. Following centrifugation (3200 × *g* for 10 min), the bacterial pellet was resuspended in 2xTY medium supplemented with kanamycin (50 mg/L), ampicillin and 0.1% w/v glucose. The resuspended cells were then incubated overnight at 30°C. The supernatant was harvested by centrifugation (3200 × *g* for 10 min) and the phage was precipitated using 20% cold polyethylene glycol 6000 and 2.5 M NaCl. The mixture was then incubated for 1 h on ice. After centrifugation, viral particles were harvested and to eliminate any residual remnants, phosphate‐buffered saline (PBS) solution was used to resuspend the resulting pellet and then centrifuged for 10 min at 11,600 × *g*.

To evaluate the recombinant fusion protein expression, an indirect enzyme‐linked immunosorbent assay (ELISA) was carried out. Briefly, a 96‐well polystyrene ELISA plate (Nunc, Roskilde, Denmark) was used to coat with either the hybrid phage or the wild‐type phage (100 µg/mL). The plate was then incubated overnight at 4°C. To eliminate any non‐specific affinities, the wells of the ELISA plate were blocked using PBS containing 3% bovine serum albumin (BSA; Sigma‐Aldrich, USA) and incubated at 37°C for 2 h. Then, as the source of the primary antibody, the anti‐HA2‐matrix protein 2 ectodomain (M2e) polyclonal antibody obtained from previously immunized rabbit was added to the wells and incubated at 37°C for 2 h. Following washing the wells using PBS with Tween 20 (PBST), the anti‐rabbit immunoglobulin G (IgG) peroxidase (Serotec, UK) was added to each well, and the plate was then incubated for 1 h at 37°C. The colour development was carried out using 3, 3′, 5, 5′ tetramethylbenzidine (TMB) substrate for 20 min. The enzymatic reaction was terminated using 1 M H_2_SO_4_, and the respective absorbance (450 nm) was calculated by an ELISA plate reader (Biotek, USA).

Furthermore, the expression of the recombinant HA2‐gVIII protein was also confirmed using Tricine sodium dodecyl‐sulphate polyacrylamide gel electrophoresis (SDS‐PAGE) as described earlier (Schägger 2006), followed by immunoblotting. The total protein was separated by electrophoresis onto a 15% polyacrylamide gel and subsequently transferred onto a nitrocellulose membrane. The resulting blots were then blocked for 1 h at 37°C using 3% BSA/PBS. The primary antibody, rabbit anti‐HA2‐M2e polyclonal antibody, was diluted (1:2000 in blocking buffer) and added, followed by incubation at 37°C for 1 h. After three washing steps, the anti‐rabbit IgG peroxidase (diluted 1:2000 in blocking buffer) was added and the plate was incubated for an additional 1 h at 37°C. The colour developed by the addition of the 4‐chloro‐1‐naphthol substrate.

### 2.5 Birds' Housing and Conditions

A total of 55 specific pathogen‐free (SPF) 1‐day‐old commercial broiler chickens were obtained from the Razi Vaccine and Serum Research Institute (Alborz, Iran). The birds were randomly allocated into 5 separate groups, each group comprised 11 birds, considering a density of 20 birds per m^2^. Given the infectious nature of the challenge agent, the birds of each treatment group were kept in separate isolated rooms under strict biosecurity conditions to prevent transmission of infectious agents. Adequate feed and water were provided ad libitum to all chickens. No additional birds were introduced or removed during the course of the experiment. All animal experiments conducted in this study were approved by the Research Ethics Committees of Faculty of Shahid Bahonar University of Kerman (Code: IR. UK.VETMED. REC. 1399.0 08).

### 2.6 Chicken Immunization and Infection

The immunogenicity of hybrid phage was evaluated through the immunization of broiler chickens. Birds were divided into five separate groups, as follows: birds vaccinated with hybrid phage and challenged (HA2‐C), birds vaccinated with commercial inactivated oil adjuvanted Poultry Influenza Vaccine (H9N2, Razi Vaccine and Serum Research Institute, Alborz, Iran) and challenged (Com‐C), birds received wild‐type phage and challenged (M13‐C), birds received PBS and challenged (NV‐C) and birds received PBS and not challenged (NV‐NC). Immunization was performed intramuscularly on Day 8 with approximately 1 × 10^10^ phage/200 µL supplemented with complete Freund's adjuvant (Sigma‐Aldrich, USA). Control birds in NV‐NC, NV‐C and M13‐C groups received the same amount of either PBS or wild‐type phage. The second vaccine dose was administered with the same volume as Dose 1, 1 week after the first dose (Day 15 of the experiment) without any adjuvant used. For Influenza disease induction, the H9N2 subtype of IAV (A/Chicken/Iran/SH‐110/H9N2) was obtained from Razi Vaccine and Serum Research Institute, Alborz, Iran. All birds, except those in group NV‐NC, were then intranasally challenged with 0.2 mL 10^6^ Embryo infectious Dose 50 (EID_50_) 1 week after the final vaccination dose (Day 22 of the experiment). NV‐NC group did not undergo any challenge inoculation.

**FIGURE 1:**
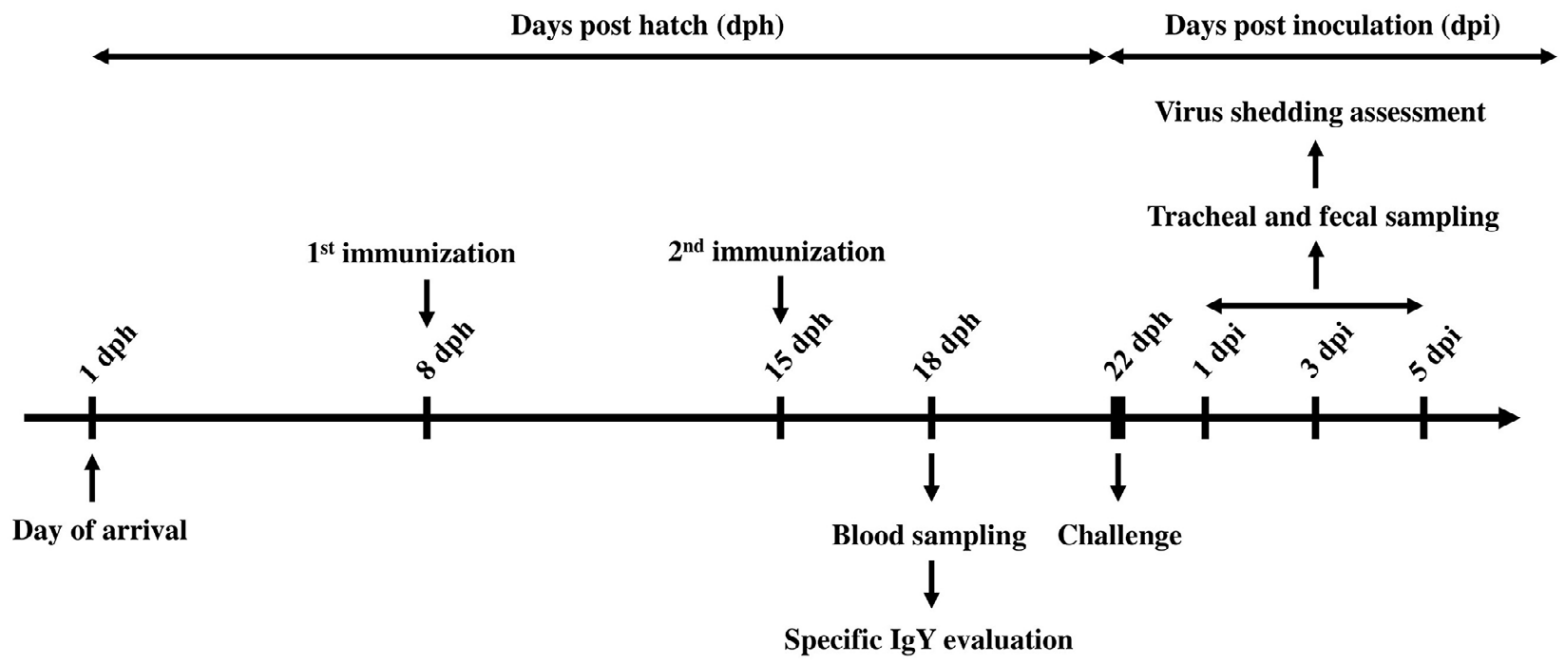
Schematic outline of the chicken experimental design.

The vaccination schedule was adapted from the manufacturer's protocol for the commercial vaccine, with minor modifications. The first dose was administered on Day 8, consistent with recommendations for broilers. A booster was given 1 week later to strengthen the primary response and enhance antibody production within the birds' short production cycle, ensuring immunity before market age. The challenge was performed on Day 22, 2 weeks after the initial vaccination, to evaluate protective efficacy at a time when immune responses were expected to be elicited. A schematic representation of the experimental design is depicted in Figure 1.

### 2.7 Sample Collection

Blood samples were collected from all birds on Day 18, obtained from the left wing vein. After centrifugation (1800 × *g*/10 min at room temperature), the sera were separated and stored at −20°C for future ELISA studies. Furthermore, tracheal and cloacal swab samples were taken from each bird using sterile cotton swabs at Days 1, 3 and 5 post‐challenge infection. Sampling was performed by inserting swabs into the cloaca and rotating them in a back‐and‐forth motion four times. The swab samples were then transferred to a liquid nitrogen storage tank and subsequently stored at −80°C for the subsequent assessment of viral shedding. To ensure consistency, all tracheal and cloacal swabs were collected by the same trained personnel following a standardized operating protocol. Identical sterile swabs were used for each bird, and sampling was performed under uniform handling conditions to minimize variability across individuals and time points. All serum and swab samples were coded prior to ELISA and viral shedding assays, thereby ensuring blinding of the investigator to group assignments and minimizing potential bias in data interpretation.

### 2.8 Measurement of Antibody Responses

The immunoglobulin Y (IgY) responses in birds vaccinated twice with the hybrid phage were assessed using an indirect ELISA. A 96‐well microtiter plate (Nunc, Roskilde, Denmark) was coated with 100 µL of hybrid phage, wild‐type phage and PBS (100 µg/mL) and the plate was incubated overnight at 4°C. Following the plates were blocked (3% BSA/PBS), the sera (1:100 diluted) obtained from immunized and control birds were added to respective wells as the source of the primary antibody. After the incubation at 37°C for 1 h, the plate was washed three times with PBST. As the secondary antibody, anti‐chicken IgY horseradish peroxidase (HRP) conjugated (Genscript, Piscataway, USA) was added and the plate was incubated for 1 h at room temperature. The colorimetric detection was performed using a TMB substrate and the final absorbance (450 nm) was determined using the plate reader.

### 2.9 RNA Extraction and cDNA Synthesis

Tracheal and cloacal swabs collected on Days 23, 25 and 27 of the experiment were processed to synthesize DNA and detect viral shedding. The High Pure RNA Tissue kit (Roche, Germany) was employed to extract total RNA from the swab samples obtained from both vaccinated and control birds, following the manufacturer's protocols. Reverse transcription of the extracted RNA (1 mg) was performed using the AccuPower CycleScript RT PreMix (dN6) kit (Bioneer, South Korea), generating cDNA. The resulting cDNA was stored at −20°C for future utilization.

### 2.10 Real‐Time PCR Assay

The evaluation of viral shedding in the trachea and cloaca of vaccinated and non‐vaccinated birds was conducted using synthesized cDNA and a quantitative real‐time PCR (qPCR) assay. The quantitative analysis was performed using the FastStart SYBR Green Master kit (Roche, Germany) in a total reaction volume of 25 µL. The reaction mixture consisted of 5.5 µL of template cDNA, 12.5 µL of RealQ Plus 2x Master Mix Green (Ampliqon, Denmark), 1.5 µL of each primer and 4 µL of dH2O. The specific primers used for amplification were as follows: forward primer, 5′‐AAGACCAATCCTGTCACCTCTGA‐3′, and reverse primer, 5′‐CAAAGCGTCTACGCTG CAGTCC‐3′. The qPCR cycling conditions included an initial denaturation step at 95°C for 10 s, followed by 40 cycles of denaturation at 95°C for 15 s, annealing at 58°C for 1 min and extension at 72°C for 1 min.

### 2.11 Statistical Analysis

The statistical analysis was conducted using GraphPad Prism 9.0 (Graph‐Pad Software, San Diego, CA). ELISA results, antibody titres of the immunized birds, and viral shedding data were analysed using one‐way or two‐way ANOVA followed by Tukey's post‐test. The data were presented as means ± SEM, and the statistical difference was assessed using a significance level of *p* < 0.05.

## 3 Results

### 3.1 Recombinant HA2 (1–9)‐gVIII pIT2 Vector Construction

**FIGURE 2:**
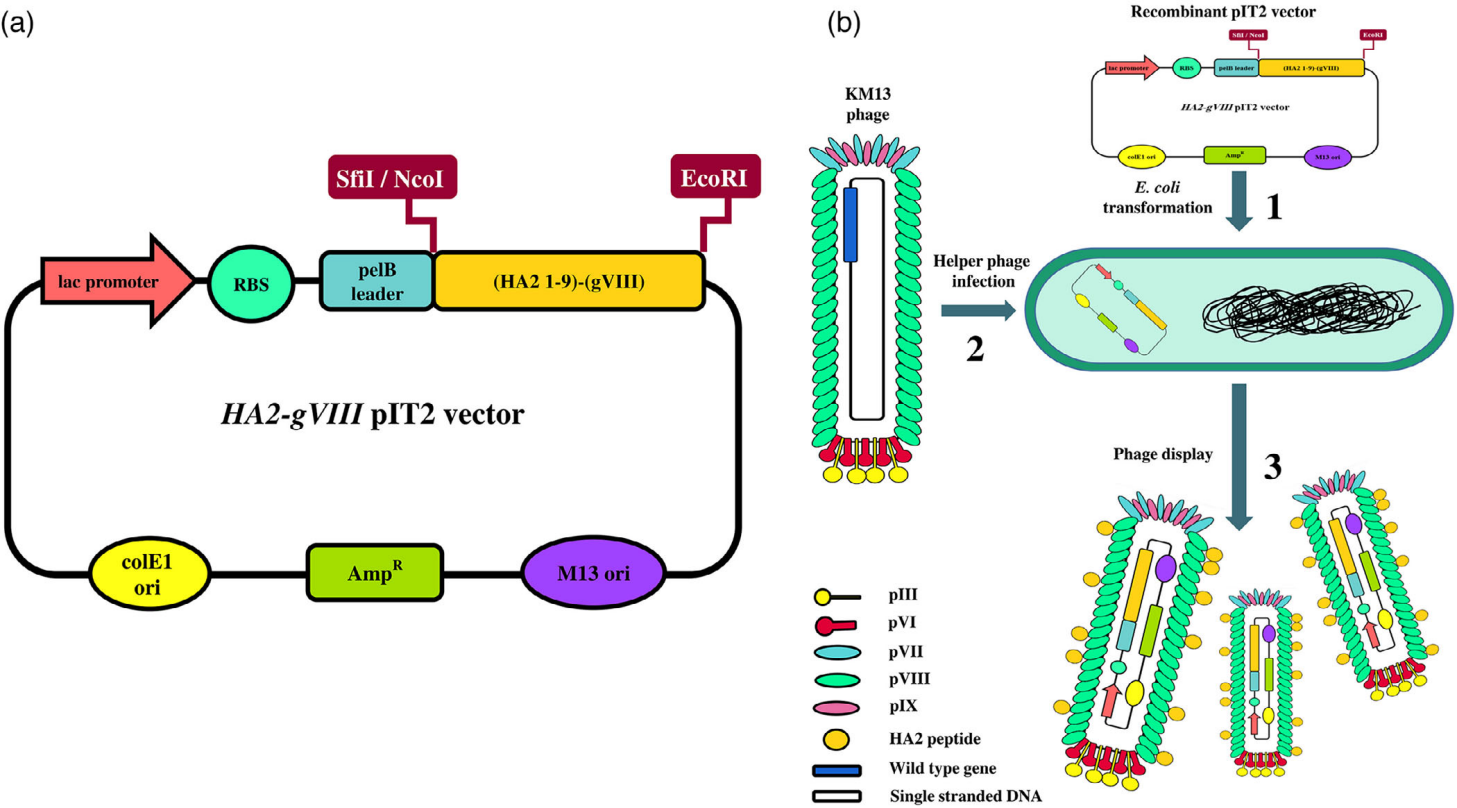
Schematic representation of the constructed HA2 (1–9)‐gVIII pIT2 vector. The HA2‐gVIII fusion gene was inserted into pIT2 phagemid vector (a), followed by introducing into *E. coli* competent cells (b, Stage 1). The transformed cells were then superinfected with the KM13 helper phage (b, Stage 2). The fusion gene was inserted into *E. coli* cells and the recombinant HA2 peptides were effectively displayed on the surface of the major coat protein pVIII of the M13 phage (b, Stage 3).

**FIGURE 3:**
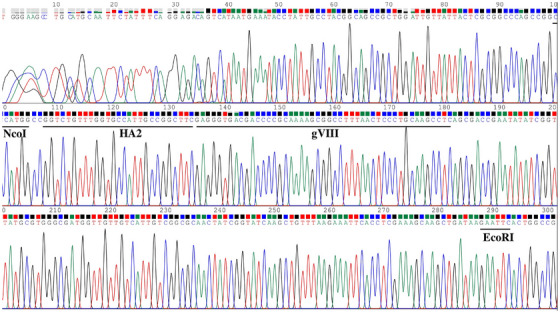
Sequencing result of the recombinant HA2 (1–9)‐gVIII pIT2 vector with LMB3 primer.

The gene encoding the HA2 peptide from the influenza virus was genetically fused with the N‐terminus of the major coat protein pVIII. The fusion gene was integrated into the pIT2 vector at the NcoI and EcoRI restriction sites, as illustrated in Figure 2a. The pIT2 phagemid vector containing the *HA2‐gVIII* fusion gene was introduced into *E. coli* cells, followed by superinfection with the KM13 helper phage (Figure 2b). A gene fragment measuring 186 bp was observed upon digestion of the recombinant HA2 (1–9)‐gVIII pIT2 vector using NcoI and EcoRI restriction enzymes (data not shown). In addition, the sequencing analysis confirmed the successful cloning of the HA2‐gVIII gene into the pIT2 vector (Figure 3).

### 3.2 Expression of Surface‐Displayed HA2 Peptide

**FIGURE 4:**
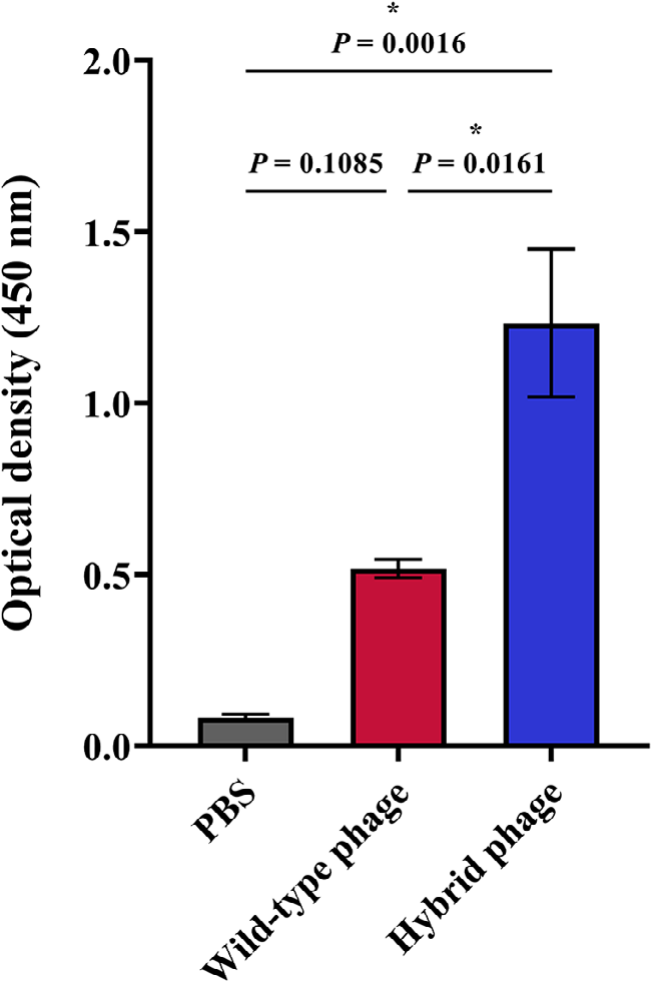
4 ELISA assay for detection of HA2 peptide (GLFGAIAGF) using anti‐HA2‐M2e polyclonal antibody. The hybrid phage (HA2 1–9‐pVIII phage) and wild‐type phage (M13 phage) were coated in ELISA plate. Anti‐HA2‐M2e polyclonal antibody and anti‐rabbit IgG peroxidase (Serotec, UK) were applied as primary and secondary antibodies, respectively. Reaction was read at 450 nm with TMB substrate. Each value represents mean ± SEM and *p* ˂ 0.05 was considered the significant level. All samples were analyzed in triplicate.

The expression of the HA2 peptide in the hybrid phage, which presented the GLFGAIAGF epitope, was evaluated using an indirect ELISA. The hybrid phage carrying GLFGAIAGF epitope represented a considerable level of HA2 peptide expression on the surface compared with M13 phage carrying the empty vector (*p* = 0.016) (Figure 4). Moreover, there was no HA2 protein expression in the wild‐type phage (*p* = 0.108).

**FIGURE 5:**
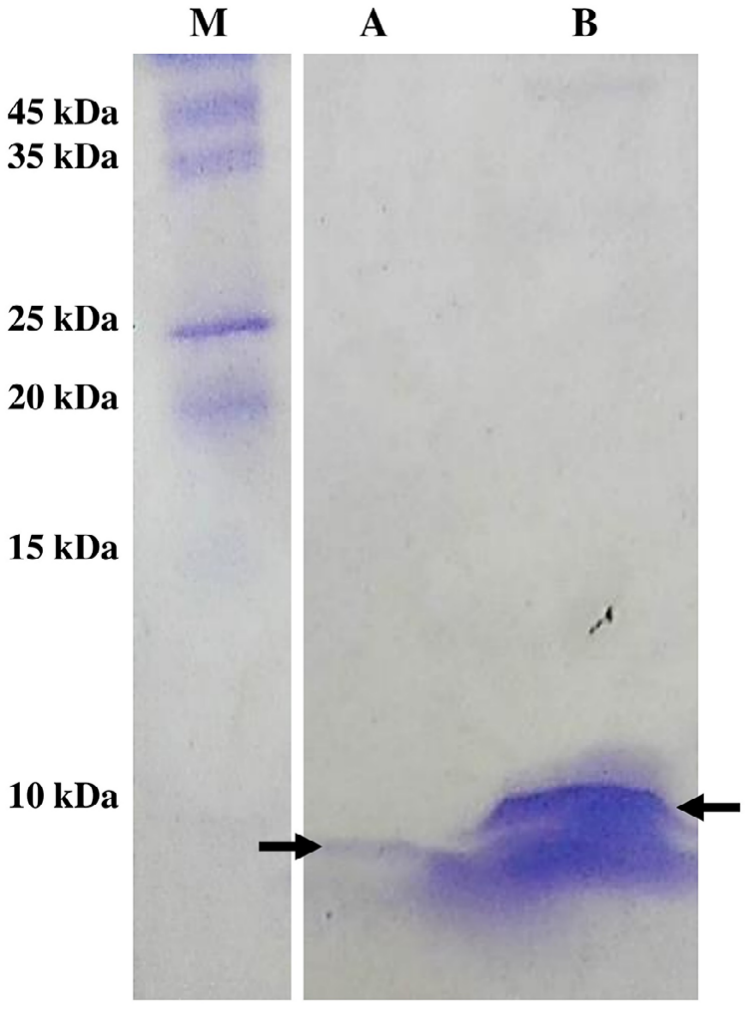
Detection of hybrid phage (HA2 1–9‐pVIII phage) and wild‐type phage (M13 phage) by Tricine‐SDS‐PAGE. Lane M: ExcelBand All Blue Broad Range Protein Marker (Smobio, Hsinchu, Taiwan); Lane A: wild‐type (M13 phage); Lane B: Hybrid phage (HA2 1–9‐pVIII phage). The rightward arrow symbols show the approximately 5‐kDa band of the wild‐type pVIII protein. The leftward arrow symbol shows hybrid capsomers with the molecular weight of 6 kDa.

The presence of the HA2 peptide on the surface of the hybrid phage was confirmed using Tricine‐SDS‐PAGE. The total protein expressed by the hybrid phage was separated on the Coomassie‐stained gel. The hybrid phage displaying the HA2 peptide exhibited two distinct bands: an approximately 5‐kDa band corresponding to the pVIII major coat protein, and a 6‐kDa band corresponding to the recombinant pVIII protein fused with the HA2 peptide (Figure 5). In contrast, the wild‐type phage displayed a single 5‐kDa band representing the pVIII major coat protein (Figure 5).

**FIGURE 6:**
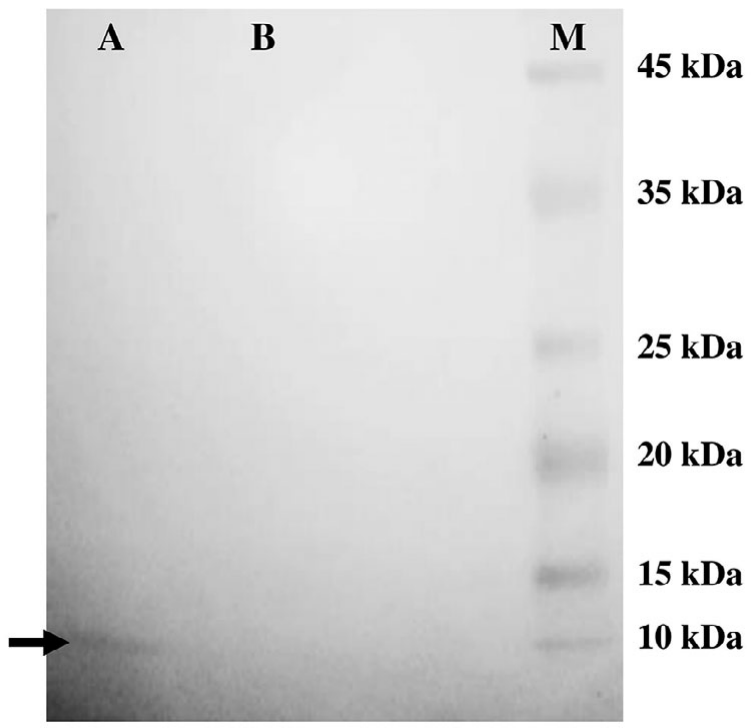
Detection of the HA2 peptide (GLFGAIAGF) using anti‐HA2‐M2e polyclonal antibody by western blotting. Hybrid phage (HA2 1–9‐pVIII phage) and wild‐type phage (M13 phage) were separated on Tricine‐SDS‐PAGE and transferred electrophoretically onto a nitrocellulose membrane. Horseradish peroxidase‐conjugated anti‐rabbit IgG (Serotec, UK) was used as the secondary antibody; Lane A: hybrid phage (HA2 1–9‐pVIII phage), Lane B: wild‐type (M13 phage); Lane M: ExcelBand All Blue Broad Range Protein Marker (Smobio, Hsinchu, Taiwan). The arrow shows the pVIII protein (approximately 6 kDa) of the hybrid phage.

Furthermore, the immunoreactivity of the HA2 peptide expressed by the hybrid phage carrying epitope GLFGAIAGF with anti‐HA2‐M2e polyclonal antibody was evaluated by immunoblotting. The HA2 peptide‐specific band was successfully detected on the nitrocellulose membrane of the hybrid phage, as depicted in Figure 6. Conversely, no distinct protein band related to the HA2 peptide was detected in the wild‐type phage (Figure 6).

### 3.3 Chicken IgY Responses

**FIGURE 7:**
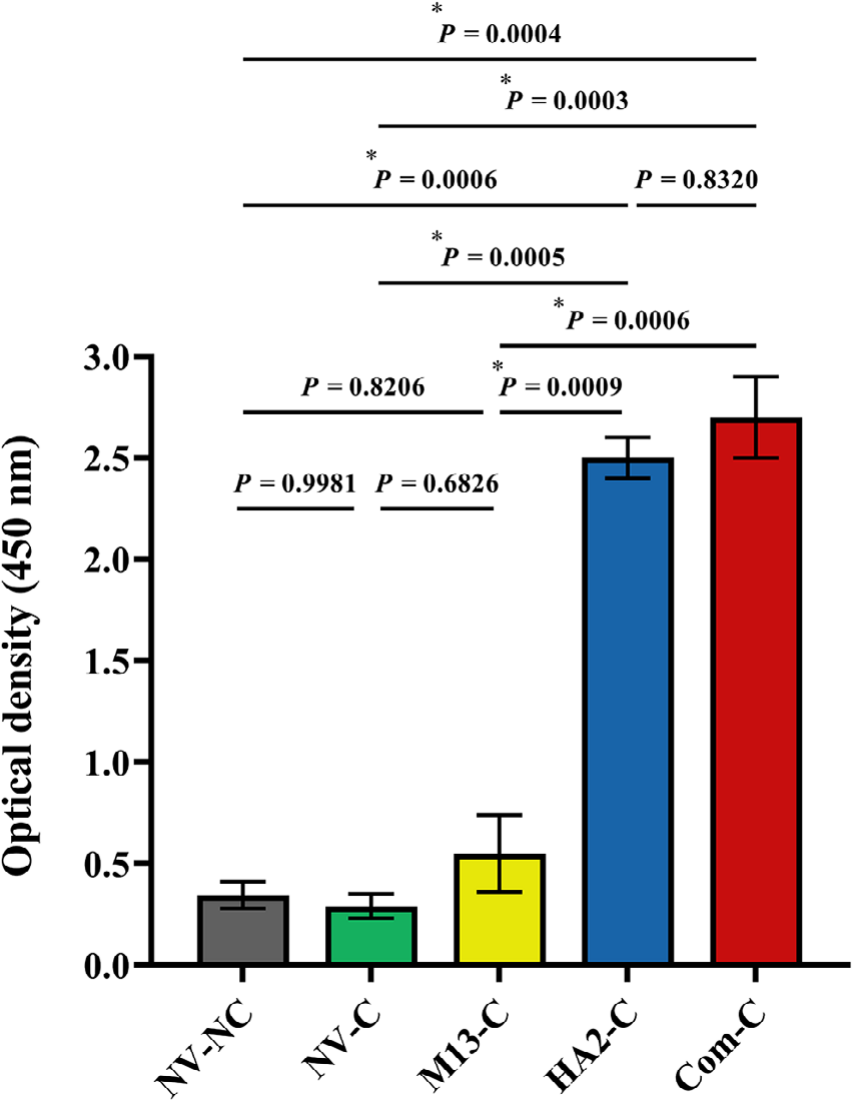
Indirect ELISA for detection of specific anti‐HA2‐gVIII polyclonal antibody in broiler chickens. The hybrid phage (HA2 1–9‐pVIII phage) was coated in ELISA plate. Sera from vaccinated and control birds were used as the source of the primary antibodies, followed by addition of anti‐IgY horseradish peroxidase conjugated (Genscript, USA) as the secondary antibodies. The color was developed using TMB substrate and the absorbance was determined at 450 nm. Values represent mean ± SEM. Asterisks show significant statistical differences (*p* ˂ 0.05). NV‐NC, not‐vaccinated and not‐challenged birds; NV‐C, not‐vaccinated but challenged birds; M13‐C, birds received wild‐type phage and challenged; HA2‐C, birds vaccinated with recombinant phage expressing HA2 peptide and challenged; Com‐C, birds vaccinated with commercial influenza vaccine and challenged. All samples were analyzed in triplicate.

The ability of the recombinant HA2 peptide to induce specific IgY antibody responses was assessed by immunizing birds intramuscularly with the hybrid phage twice, on Days 8 and 15 of the experiment. Serum antibody responses of all chickens were measured using indirect ELISA, 3 days after the final vaccination (Day 18 of the experiment). As shown in Figure 7, chickens immunized with the hybrid phage displaying HA2 peptide exhibited significantly higher specific IgY responses compared with control birds (*p* < 0.05). Likewise, the commercial H9N2 vaccine elicited significant specific IgY responses compared with those in NV‐NC and NV‐C groups (*p* < 0.05). There was no considerable statistical difference in the level of IgY responses between birds in the Com‐C and HA2‐C groups (*p* = 0.8320). In addition, unvaccinated birds, either challenged or not challenged, and those received wild‐type phage did not induce any significant antibody responses (*p* > 0.05).

### 3.4 Virus Shedding Assessment

**FIGURE 8:**
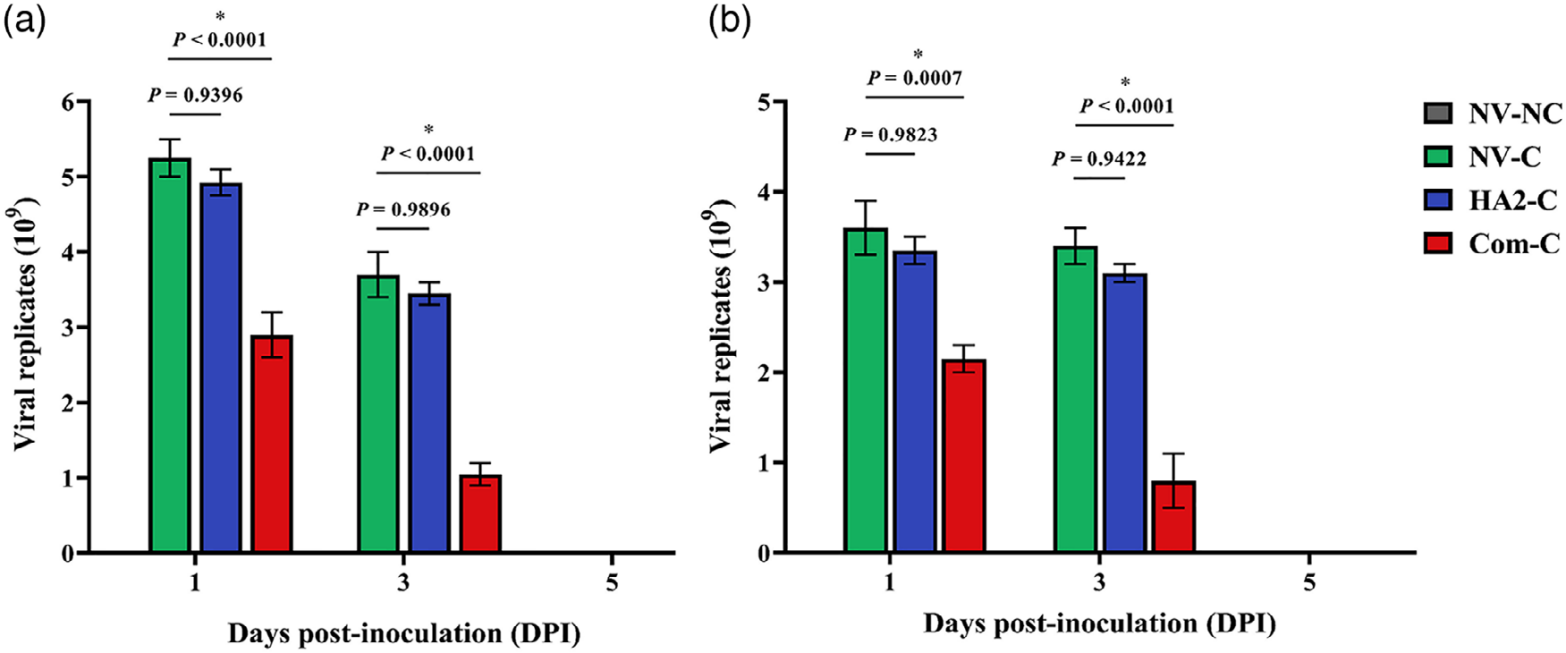
Shedding of H9N2 virus in the trachea and cloaca of chickens. Tracheal and cloacal swab samples were collected on Days 23, 25 and 27 of the experiment from vaccinated and control birds to determine the efficacy of the recombinant M13 phage carrying HA2‐peptide in reducing the number of the challenge H9N2 virus replicates in the trachea (a) and cloaca (b). Each value represents mean ± SEM. Asterisks show significant differences (*p* ˂ 0.05). NV‐NC, not‐vaccinated and not‐challenged birds; NV‐C, not‐vaccinated but challenged birds; HA2‐C, birds vaccinated with recombinant phage expressing HA2 peptide and challenged; Com‐C, birds vaccinated with commercial influenza vaccine and challenged.

The replication levels of the H9N2 subtype virus in the trachea and cloaca were assessed to evaluate viral shedding. Real‐time PCR was used to quantify the replication level at 1, 3 and 5 days after challenge in both vaccinated and control birds. The birds vaccinated with the hybrid phage expressing recombinant HA2 peptide did not show a significant reduction in viral shedding in the trachea and cloaca at Days 1 or 3 post‐inoculation compared with the NV‐C control group (*p* > 0.05) (Figure 8). However, birds immunized with the commercial influenza vaccine exhibited a statistically significant decrease in tracheal and cloacal shedding of virus at 1‐ and 3‐days post‐challenge (*p* < 0.05) (Figure 8). The NV‐NC control birds did not show any level of virus replication either in trachea or cloaca. Moreover, on Day 5 after challenge inoculation, there were no levels of virus replication in all vaccinated and control groups, both in the trachea and cloaca.

## 4 Discussion

The persistent emergence of novel IAVs strains continues to pose a significant public health challenge, contributing to the burden of respiratory illnesses and increasing the risk of global pandemics (Margine et al. 2013; Varečková et al. 2013). Hence, adopting preventive measures that focus on the conserved and immunogenic regions of the causative agent could serve as a crucial strategy for effectively controlling and managing Influenza disease, while circumventing concerns related to the emergence of novel strains. The HA2 protein of the influenza virus contains two immunogenic regions (To et al. 2015). One of the regions within the HA2 segment (Position 26–40) exhibits immunogenic properties that overlap with the heptad repeat region (Position 34–79), while the other region, consisting of the N‐terminal 1–9 amino acid residues, demonstrates a high level of conservation across various subtypes of IAVs (Du et al. 2010; To et al. 2015). Being highly conserved across all known subtypes of IAVs, the N‐terminal fragment of HA2 represents an attractive candidate for universal influenza vaccine development (Du et al. 2010; Staneková and Varečková 2010). Previous studies have demonstrated that this conserved sequence can elicit broadly reactive immune responses and confer cross‐protection against heterologous influenza virus strains (Varečková et al. 2008; Stropkovská et al. 2009; Prabhu et al. 2009). In the present study, we utilized this region as a promising target to evaluate its potential in vaccine development against IAVs. However, due to its small size, the region requires chemical or genetic coupling with larger carriers in order to augment its immunogenicity (Lamb et al. 1985; Feng et al. 2006; Bachmann and Jennings 2010). Previous investigations enhance the immunogenicity of HA2 peptide derived from the influenza virus by employing multiple protein carriers including GST (Golchin et al. 2018), IVL (S. Chen et al. 2015), norovirus P and adenovirus (Gong et al. 2016) and *L. plantarum* (Yang et al. 2017, Yang et al. 2018; Bo et al. 2019).

In recent years, there has been an increasing fascination with the use of phages as protein carriers displaying the respective antigens for therapeutic, diagnostic and vaccine development purposes (Ma et al. 2019; Xue et al. 2019; W. Chen et al. 2021; Sun et al. 2021; Grabowski et al. 2023). In the current study, we used a phage system based on the filamentous bacteriophage M13, formerly recognized as one of the widely employed bacteriophages (Grabowski et al. 2023), to display the respective peptide of the influenza virus. In order to accomplish this objective, we employed genetic fusion methodologies to conjoin the first nine amino acids of HA2 with the N‐terminal segment of the principal protein found in the coat of the M13 phage. This fusion strategy enabled the development of a robust peptide immunogen. Importantly, by leveraging the surface expression capabilities of filamentous phages, small peptides can be effectively presented as part of phage proteins, thereby augmenting their immunogenic potency (Lamb et al. 1985; Bachmann and Jennings 2010). The efficient amplification of filamentous phages within susceptible *E. coli* cell cultures, coupled with their ability to rapidly generate diverse libraries of phages displaying various peptides on their surfaces, signifies advantageous aspects of utilizing phages as a display system (Grabowski et al. 2023). The M13 phage possesses a notable attribute of physical stability, rendering it an advantageous platform. Through a mechanism of multiple displays, the phage particle is able to effectively present small peptides on its surface, facilitated by the presence of 2700 pVIII protein copies (Harder and Vahlenkamp 2010).

Several studies have reported that the assembly of phage particles and the recombinant pVIII protein copy numbers in hybrid phages are significantly influenced by the size of heterologous peptides (Rowe et al. 1999). In our study, we selectively fused a mere nine amino acids from the HA2 region (1–9) to the N‐terminal fragment of the pVIII protein within the M13 phage. As the HA2 antigen is not displayed on all pVIII coat proteins (Greenwood et al. 1991, Malik et al. 1996), the SDS‐PAGE analysis of the hybrid phage showed two protein bands: one corresponding to the pVIII major coat protein (approximately 5 kDa), and a higher molecular weight band representing pVIII coat proteins fused with the HA2 peptide (6 kDa). This finding aligns with prior research investigating phages that display epitopes (Du et al. 2010).

The surface display of foreign immunogens on host cells has been widely employed in previous research as a strategy to develop vaccine candidates targeting various infectious diseases (Ju et al. 2021; Shamshirgaran et al. 2022, Shamshirgaran et al. 2023; Huynh et al. 2023), especially avian influenza (Jiang et al. 2017; Yang et al. 2018; Bo et al. 2019; Niu et al. 2021). Formerly, various display platform technologies including bacterial display systems such as *L. plantarum* (Jiang et al. 2017; Yang et al. 2017; Bo et al. 2019; Niu et al. 2021), as well as yeast display systems like *S. cerevisiae* (Lei et al. 2021; Xie et al. 2023; H. Zhang, Li, et al. 2023; H. Zhang, Xie, et al. 2023), were employed for surface expression of immunogens in vaccine candidates against influenza virus. However, phage display platforms outperform bacterial and yeast display systems by exhibiting superior surface expression capacity for peptides, generating a broader range of diverse peptide variants and clones and offering a less complex and more streamlined library preparation process (Grabowski et al. 2023). In the present study, we employed a phage display platform to express HA2 peptide on the surface of a recombinant filamentous M13 phage, serving as a vaccine candidate against the H9N2 influenza virus challenge in broiler chickens.

Several studies have indicated that the surface expression of the HA antigen of the influenza virus holds promise as an effective strategy for achieving high protein yield. This is further demonstrated by the potential of the respective surface‐displayed antigens in providing protection for animal models against challenge infections. The HA antigen has been employed in vaccine investigations either independently or in conjunction with other immunogens from the influenza virus. The surface display of the HA peptide on the *L. plantarum* (Jiang et al. 2017; Yang et al. 2018; Niu et al. 2021) or *Lactobacillus delbrueckii* (Z. Wang et al. 2013) holds considerable potential in bolstering the immune responses of vaccinated birds, surpassing those observed in non‐immunized chickens and also in providing protection against experimental Influenza infection. The vaccinated birds exhibited robust levels of cellular, humoral and mucosal responses, highlighting the efficacy of this approach in evoking the immune system. Moreover, a vaccine strain, *L. plantarum* expressing HA and NA antigens, elicited specific intestinal immunoglobulin A (IgA) and serum IgG responses in vaccinated mice and also conferred significant protection against the infectious experiment (Bo et al. 2019). The surface display of the fused HA and M2e antigens has also exhibited promising results in augmenting systemic and mucosal immunity and conferring protection in birds against experimental influenza virus challenge (Yang et al. 2018). In a related study by To et al. (2015) a fusion peptide (GLFGAIAGF, residues 1–9) derived from HA2, which showed antigenic conservation, was tested in a mouse model. This fusion peptide provided complete protection against highly pathogenic avian Influenza H5N1 and H7N9 viruses from different clades (To et al. 2015). Furthermore, Golchin et al. (2018) expressed the M2e‐HA2 sequence of the influenza virus as a fusion protein and demonstrated its protective effect against H9N2 virus infection in mice (Golchin et al. 2018). The outcomes of these investigations using the HA antigen represented both the significant protein expression and the elicitation of protective immune responses in immunized animal models. Accordingly, these findings highlight the significant potential of surface expression systems in vaccine development. In line with these vaccine studies, we confirmed the successful surface expression of the HA2 (1–9) peptide on the hybrid phage through immunoblotting and ELISA. The western blot analysis revealed an immune‐reactive band with slightly slower migration compared to the wild‐type phage, indicating specific detection of the desired peptide. Furthermore, the immunogenicity assessment of the hybrid phage demonstrated its ability to induce high levels of IgY antibodies specific to HA2 after intramuscular immunization in broiler chickens.

Nevertheless, the vaccine candidate developed in this study did not demonstrate sufficient efficacy in reducing viral shedding in the trachea and cloaca of vaccinated birds. Viral shedding is a key parameter for evaluating the efficacy of vaccination against IAVs. Reductions in the levels and duration of shedding are indicative of vaccine potential in limiting the infectious disease (Spackman et al. 2016, Kapczynski et al. 2016). Being used as the indicator of the effective inoculation, viral shedding could also reflect the transmission potential (Germeraad et al. 2019). Importantly, cloacal shedding has been identified as the critical contributor to sustaining a chain of transmission (Ruiz‐Hernandez et al. 2016). This indicated that even when systemic antibody responses are induced, continued virus shedding from infected birds may facilitate virus transmission within and between flocks, which could compromise the flock immunity (Ruiz‐Hernandez et al. 2016; Germeraad et al. 2019). The absence of a significant reduction in viral shedding in this study may be attributed to several contributing factors. One of the primary considerations in this study is the immunization protocol, which involved two intramuscular vaccine doses administered one week apart. This short interval between doses may not have allowed sufficient time for the immune system to mount a fully protective response. Previous studies have shown that administering two intramuscular doses with a longer interval, such as two weeks, is more effective in reducing viral shedding in birds vaccinated against IAV (Kim et al. 2017). Moreover, the limited reduction in shedding could also reflect insufficient mucosal immune responses, which were not assessed in this study and remain a critical area for future research. Parenteral vaccination primarily stimulates systemic immunity, whereas mucosal vaccination strategies, such as intranasal or oral delivery, have been shown to elicit stronger mucosal immune responses in poultry against IAV and other infectious diseases (Wells and Mercenier 2008; R. Zhang, Xu, et al. 2023; Shamshirgaran and Golchin 2025b). Therefore, employing other delivery routes, such as oral or intranasal administration, may enhance the efficacy of the vaccine evaluated in this study. In addition, the phage display system employed in this study presented only a single antigen, HA2. Although this strategy demonstrated partial efficacy, multivalent approaches incorporating additional conserved antigens, such as M2e or NA, have been reported to significantly reduce viral titres in the respiratory tract and confer broader protective immunity in birds (Yang et al. 2018; Hajam et al. 2020). Future studies should therefore explore optimized immunization regimens, alternative delivery routes and combinatorial antigen strategies, which may enhance the capacity of phage‐based vaccines to limit viral shedding and transmission.

In conclusion, we have successfully engineered a filamentous hybrid phage capable of carrying the GLFGAIAGF epitopes of the IAVs and displaying the HA2 peptide on its surface. The respective phage elicited the specific serum IgY antibodies in vaccinated birds against the HA2 protein, which is present in all subtypes of IAVs. While the hybrid phage exhibits promising potential as a candidate for antibody production, its failure to reduce the level of viral shedding in the tracheal secretion and cloacal samples emphasizes the need for more extensive investigation to thoroughly assess its protective efficacy and practical utility.

## Author Contributions

**Zinat Lotfi**: conceptualization, data curation, investigation, writing – original draft preparation. **Mehdi Golchin**: conceptualization, methodology, validation, resources, project administration, writing – review and editing. **Mohammad Ali Shamshirgaran**: conceptualization, methodology, visualization, validation, data curation, supervision, formal analysis, writing – original draft preparation, writing – review and editing. All authors have thoroughly reviewed and approved the final manuscript.

## Funding

The authors have nothing to report.

## Ethics Statement

All animal experiments conducted in this study underwent rigorous assessment and received approval from the Research Ethics Committees of the Faculty of Shahid Bahonar University of Kerman (Code: IR. UK. VETMED. REC.1399.0 08).

## Conflicts of Interest

The authors declare no conflicts of interest.

## Data Availability

All data generated or analysed during this study are included in this published article.
